# Sprint Start Kinetics of Amputee and Non-Amputee Sprinters

**DOI:** 10.1371/journal.pone.0166219

**Published:** 2016-11-15

**Authors:** Steffen Willwacher, Volker Herrmann, Kai Heinrich, Johannes Funken, Gerda Strutzenberger, Jan-Peter Goldmann, Björn Braunstein, Adam Brazil, Gareth Irwin, Wolfgang Potthast, Gert-Peter Brüggemann

**Affiliations:** 1 Institute of Biomechanics and Orthopaedics, German Sport University Cologne, Cologne, Germany; 2 School of Sport, Cardiff Metropolitan University, Cardiff, Wales; 3 Department of Sport Science and Kinesiology, University of Salzburg, Salzburg, Austria; 4 German Research Centre of Elite Sport, German Sport University Cologne, Cologne, Germany; University of France Comté, FRANCE

## Abstract

The purpose of this study was to explore the relationship between the forces applied to the starting blocks and the start performances (SPs) of amputee sprinters (ASs) and non-amputee sprinters (NASs). SPs of 154 male and female NASs (100-m personal records [PRs], 9.58–14.00 s) and 7 male ASs (3 unilateral above knee, 3 unilateral below knee, 1 bilateral below knee; 100 m PRs, 11.70–12.70 s) with running specific prostheses (RSPs) were analysed during full-effort sprint starts using instrumented starting blocks that measured the applied forces in 3D. Using the NAS dataset and a combination of factor analysis and multiple regression techniques, we explored the relationship between force characteristics and SP (quantified by normalized average horizontal block power). Start kinetics were subsequently compared between ASs and NASs who were matched based on their absolute 100 m PR and their 100 m PR relative to the world record in their starting class. In NASs, 86% of the variance in SP was shared with five latent factors on which measured parameters related to force application to the rear and front blocks and the respective push-off directions in the sagittal plane of motion were loaded. Mediolateral force application had little influence on SP. The SP of ASs was significantly reduced compared to that of NASs matched on the basis of relative 100-m PR (−33.8%; *d* = 2.11, *p* < 0.001), while a non-significant performance reduction was observed when absolute 100-m PRs were used (−17.7%; *d* = 0.79, *p* = 0.09). These results are at least partially explained by the fact that force application to the rear block was clearly impaired in the affected legs of ASs.

## Introduction

Get ready, set, go! The start of the 100 m final is one of the most anticipated moments of any major athletics championship. The importance of an athlete’s start performance (SP) is inversely related to the length of the track event. It is therefore very important in the 100 m, less so in the 200 m and potentially most significant in 60 m indoor events. Despite the fact that, for a typical 100-m race, the start (including reaction time) only takes up about 5% of the total duration [1], around one third of the athlete’s maximal velocity is generated during push-off from the blocks [2]. As a result, average centre-of-mass (CoM) acceleration is highest during this phase of the race.

Following Newton’s second law of motion, horizontal CoM acceleration requires net propulsive forces to be applied to the body of the athlete in the running direction. If force application is accompanied by motion of the sprinter, mechanical work is performed. Completing a given quantity of work in less time corresponds to an increase in average power generation over that period, so this parameter is considered an excellent descriptor of SP in sprinters [3]. Muscle tissue is capable of converting metabolic energy into mechanical work at high rates during contraction [4], which makes muscle–fascicle contraction crucial for developing high CoM acceleration from a resting position. Elastic components of the muscle tendon units, but also elastic materials utilized in the dedicated running-specific prostheses (RSPs) of amputee sprinters (ASs) can store and return energy. However, they cannot increase the potential or kinetic energy of the sprinter from rest unless they have been pre-loaded by means of co-contraction prior to the initiation of the acceleration task. Given the relatively small forces applied to the blocks in the set position, pre-loading amplitudes are relatively low when compared to the forces exerted during the push-off phase [5]. Therefore, while the efficient energy storage and return provided by RSPs is beneficial in longer events like the 400 m where a high level of running economy is required [6], it seems theoretically improbable that they would allow ASs to achieve the levels of performance seen in top NASs during the sprint start. In line with this hypothesis, Taboga, Grabowski, di Prampero, & Kram [7] found that, during the block phase, unilateral below-knee, mostly sub-elite amputees performed worse than performance-matched NASs. Nevertheless, the current literature lacks empirical evidence for reduced SP in ASs at the elite level, as well as for athletes with bilateral or transfemoral amputations. Furthermore, detailed descriptions of the mechanisms underlying impaired SP in ASs are also lacking.

It is generally accepted that good acceleration performance in sprinting tasks requires highly efficient application of horizontal force [8, 9] in order to increase horizontal impulses generated during ground-contact phases. In addition, good acceleration performance requires high extension moments and positive power output by lower extremity joints in the start and early acceleration phase, particularly at the hip, knee and ankle joint [5, 10–12].

The aforementioned references indicate that acceleration performance can be improved by increasing the capacity of the musculoskeletal system to create power from a resting position. Furthermore, they show that the efficiency of horizontal force application might play an important role in improving acceleration during the start phase [8,9]. The ability to direct a great amount of the total force in the running direction can be considered a key technical skill that determines the quality of a sprinter’s starting technique. Currently, it is not clear whether the capacity for high leg power output and that for efficient direction of forces in the running direction are independent abilities that could be worked on separately, or whether both are simultaneously influenced by an underlying “acceleration ability” factor.

A deeper understanding of the mechanism underlying sprint start performance should improve sprint performance diagnostics and aid the design of technical drills and strength and conditioning programs for NASs and ASs.

Therefore, in the present study we first explored potential latent factors (determined by exploratory factor analysis [EPA]; see methods section for details) influencing ground-force application during the sprint start, and how such factors might relate to start performance in NASs. Based on the literature it was hypothesized that at least two latent factors affect force application to the blocks: One was the overall resultant force the athlete applies to them and the other was the direction of that force. Based on the enhanced understanding provided by this initial part of our study, we then compared the start performance and ground-force application characteristics of ASs and NASs.

## Methods

### Participants

Our study sample included 154 NASs at a wide range of 100-m sprint performance levels (100 m PRs, 9.58 s– 14.00 s). This NAS group comprised 103 males (mean age, 20.8 ± 3.7 years; mean body mass, 74.8 ± 7.5 kg; mean standing height, 1.81 ± 0.06 m) and 51 females (mean age: 20.0 ± 3.6 years; mean body mass, 60.8 ± 5.6 kg; mean standing height, 1.71 ± 0.06 m). The remainder of the study sample consisted of seven male ASs (see Table 1 for physical characteristics and PRs). All 100 m PR times were achieved prior to data collection, but not necessarily within the same competitive season. In unilateral amputee athletes (n = 6), body height was determined while standing on the unaffected leg. For the bilateral amputee (n = 1), standing height was measured while wearing his sprinting prostheses and leaning against a wall in order to maintain a stable standing position.

**Table 1:** Physical characteristics and personal records (PRs) of amputee sprinters.

	Amputation		Affected	Height	Mass	Age	100 m PR	Rel. 100 m PR
	level		leg	(m)	(kg)	(years)	(s)	(% WR time)
AMS01	UL	TF	right	1.89	73.8	32	12.70	105.9
AMS02	UL	TF	left	1.78	71.0	31	12.26	101.2
AMS03	UL	TF	left	1.81	80.2	30	12.40	102.4
AMS04	UL	TT	right	2.00	85.7	33	12.40	116.9
AMS05	UL	TT	right	1.91	74.7	25	11.92	112.4
AMS06	UL	TT	right	1.97	89.1	24	11.70	110.3
AMS07	BI	TT	both	1.87	69.7	27	12.27	116.1

UL = unilateral amputation; BI = bilateral amputation; TF = transfemoral amputation; TT = transtibial amputation

Written informed consent was obtained from all participants and the experimental procedures were in line with the guidelines stated in the Declaration of Helsinki. Approval was obtained from the ethical committee of the German Sport University, Cologne, Germany.

### Experimental setup and data reduction

**Fig 1:**
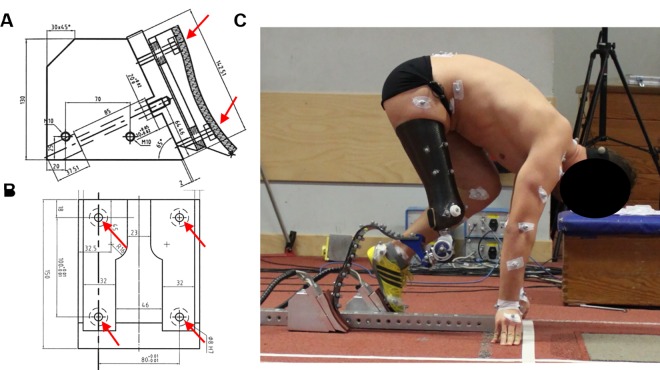
Instrumented starting blocks used in the study. A and B show schematic drawings of the blocks (including four 3D piezo-type force sensors marked by red arrows) from the side (A) and from the front (B). In C, one of the unilateral transfemoral amputees is shown in the set position.

To obtain the force data, we used a custom-made instrumented starting block consisting of a very stiff steel centre rail and separate block bases and force sensing units for each foot. Base units were available for different inclination angles and were screwed to the centre rail in order to provide a sufficiently stiff system for the force measurements, while enabling adjustment of start-block settings to those used for training and competition. Small custom-made force platforms, each including four piezo-type 3D force transducers (Kistler AG, Winterthur, Switzerland), were screwed onto the tops of the block bases for force measurements (Fig 1). Analog force signals were converted to digital at a sampling rate of 10,000 Hz. Further details of the instrumented starting blocks are provided in reference [13]. Force signals were filtered using a recursive 4^th^ order digital Butterworth filter (120 Hz cut-off frequency). Force signals were transformed from the local (tilted) starting-block reference system to a global coordinate system before further analysis was performed. The orientation of the global coordinate system was as follows: The x-axis pointed forward along the running surface (horizontal plane), the y-axis pointed to the left along the same surface plane and the z-axis pointed vertically upwards. Mediolateral forces were described as follows: Positive values were used if the block reaction forces were in the direction of the contralateral leg, and negative values corresponded to the opposite situation. Because the dominant component of force was positive in the front leg and negative in the rear leg, maximal positive values in the front leg and minimal values in the rear leg were considered the maximum mediolateral forces applied to the blocks.

The following parameters were extracted for analysis: Overall start performance was described using normalized average horizontal (in the running direction) block power (*NAHBP*) [3]. Average horizontal block power was defined as the change in horizontal kinetic energy during push-off from the blocks (*T*_*Block*_):

$$\bar{P}=\frac{m(V_{f}^{2}-V_{i}^{2})}{2T_{Block}}$$ (1)

We measured block time (*T*_*Block*_, time from first reaction to block clearance) and CoM velocity at block clearance (*V*_*f*_, determined by integration of mass-normalized horizontal force curves with initial velocity equalling zero). Body mass (m) included the mass of the prosthetic parts in ASs. As the initial velocity (*V*_*i*_) is zero in the set position of the sprint start, the formula for the calculation of average horizontal block power ($\bar{P}$) can be simplified by omitting the $V_{i}^{2}$ term:

$$\bar{P}=\frac{mV_{f}^{2}}{2T_{Block}}$$ (2)

Because athletes with different body masses and dimensions require different average powers to translate their CoM to the same extent, average horizontal block power was further normalized to body mass (*m*) and body height (*h*) in order to achieve a dimensionless normalized average horizontal block power (*NAHBP*; [14], corrected in [3]):

$$NAHBP=\frac{\bar{P}}{mg^{\frac{3}{2}}h^{\frac{1}{2}}}$$ (3)

Inserting Eq 2 into Eq 3 yields:

$$NAHBP=\frac{V_{f}^{2}}{2T_{Block}\;g^{\frac{3}{2}}\;h^{\frac{1}{2}}}$$ (4)

In contrast to the approach taken by Bezodis et al. [3], body height was used for normalisation instead of leg length, since leg length could not be obtained from all participants. To describe the force application on the starting blocks we determined average forces and impulses of the front and rear leg in antero–posterior, mediolateral, vertical and resultant directions. First reaction (i.e. the start of the push-off phase) was determined as the first instant when the resultant force curves rose from the baseline force in the set position. Block clearance was defined as the first instant when the resultant force of the front block dropped below a threshold of 50 N. To specify the efficiency of force application to the blocks, the ratio of horizontal (in the running direction) to resultant block reaction force impulse of both legs (RHRI, [8]) and the ratio of mediolateral to resultant block reaction force impulses (RMLRI) were calculated.

### Statistics

Each athlete performed at least three full-effort sprint starts over a distance of 20 m. The best start (based on NAHBP) was selected for further analysis. To identify potential latent factors affecting SP, we performed an exploratory factor analysis (EFA) using selected force parameters. EFA is a statistical procedure used to analyze variability among measured correlated variables with respect to a potentially lower number of unobserved (unmeasured) or latent variables, which are termed factors. For example, it could be the case that variations in a great number of observed sprint-start kinetic parameters are actually just the result of variability in a much smaller group of underlying parameters (factors) that represent more fundamental sprint-start abilities. EFA is aimed at finding measured parameters that vary as a group in response to latent variables. In our case, the dataset representing the observed variables included average and peak forces in all directions and parameters describing the push-off direction (RHRI, RMLRI). Using Matlab’s (R2015b; Mathworks, Natick, MA, USA) built-in “factoran” function, we calculated the maximum likelihood estimate (MLE) of the factor loadings matrix *Λ* in the factor-analysis model,

x=μ+Λf+e;

where *x* is a vector of observed force parameters, *μ* is a constant vector of means, *Λ* is a matrix of factor loadings, *f* is a vector of independent, standardized common factors, and *e* is a vector of independent specific factors. To identify the number of factors to extract for further analysis, the Kaiser criterion [15] and the scree test [16] were applied. In a subsequent step, factor loadings and scores were rotated using the “varimax” method [17] in order to improve interpretability. To identify the relationship between these latent factors and SP (NAHBP), multiple linear regression was performed with latent factor scores as the predictors and the NAHBP as dependent variable in an approach similar to Basilevsky [18]. Using forward selection, models including intercept and quadratic terms were fitted using Matlab’s “fitlm” function. The best model was determined by the Akaike information criterion [19]. Further regression analyses were performed to identify the relationships between force-application parameters, SP and 100 m PRs. For the identification of differences between ASs and NASs, two different approaches were taken. In the first, ASs were matched to NASs with similar absolute PRs, and in the second, athletes were matched with respect to their relative PRs (relative to the current world record in their particular starting class, based on International Paralympic Committee classification rules). The matching procedure was aimed at finding the three closest absolute or relative 100 m PRs for each athlete. If a NAS matched with more than one amputee athlete, s/he was only included once. Comparison between ASs and matching NASs were performed using independent-samples *t* tests. To address potential problems due to unequal variances, Satterthwaite's approximation for the effective degrees of freedom was used [20, 21]. The significance level, *α*, was set at 0.05. Due to the low size in the AS sample and related statistical power, we also calculated effect sizes (Cohens d) in order to allow for an estimation of the strength of an observed difference [22]. Effect sizes greater than 0.2 were considered small, greater 0.5 medium and greater than 0.8 large [22].

## Results

**Fig 2:**
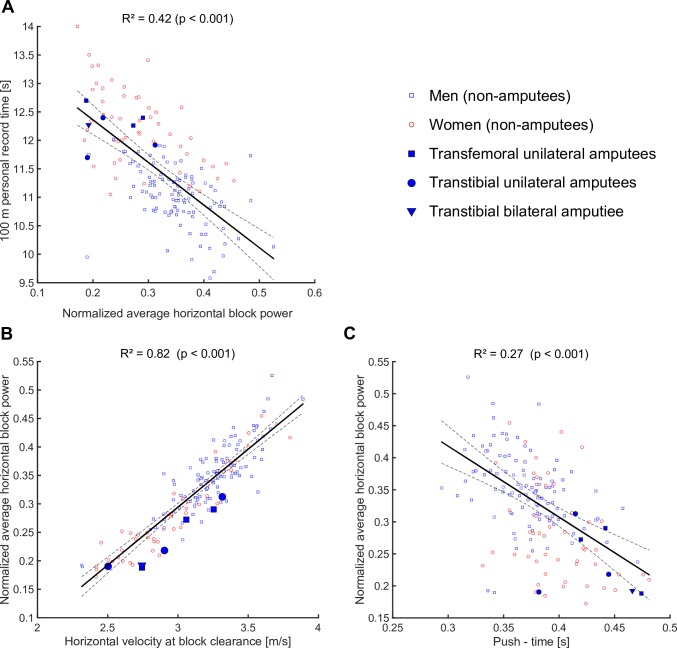
Scatter plots including fitted linear regression lines. Regression models were fitted using the non-amputee (NAS) data only. Regression lines are plotted along with broken lines that represent the 95% confidence interval.

SP shared 42% of its variance with 100 m PR in the NAS group (Table 2, Fig 2). Combined, block time and horizontal CoM velocity at block clearance predicted 98% of the variance of the start performance (NAHBP) in a multiple linear regression model, while horizontal CoM velocity and block time respectively shared 82% and 27% of their variance with NAHBP in separately performed, simple linear regression analyses (Table 2, Fig 2).

**Table 2:** Results of linear regression analyses.

			95% confidence		
Model		Coefficients	interval	t-value	p-value
response:	100 m PR				
predictor(s):	NAHBP	-7.486	[-8.896 -6.076]	-10.500	<0.001
Adj. R² = 0.42					
response:	NAHBP				
predictor(s):	Push Time	-0.811	[-0.859 -0.762]	-33.070	<0.001
	Horizontal Velocity	0.191	[0.185 0.196]	71.281	<0.001
Adj. R² = 0.98					
response:	NAHBP				
predictor(s):	Push Time	-1.030	[-1.395 -0.822]	-7.084	<0.001
Adj. R² = 0.27					
response:	NAHBP				
predictor(s):	Horizontal Velocity	0.204	[0.188 0.219]	26.097	<0.001
Adj. R² = 0.82					
response:	NAHBP				
predictor(s):	F1 - Rear leg - force sagittal	0.040	[0.036 0.045]	19.158	<0.001
	F2 - Front leg - ml force + direction	0.005	[0.001 0.009]	2.365	0.019
	F3 - Front leg - push-off direction	0.033	[0.028 0.037]	15.461	<0.001
	F4 - Front leg - max force sagittal	0.029	[0.025 0.034]	14.008	<0.001
	F5 - Rear leg - ml force + direction	-0.004	[-0.008 0.001]	-1.670	0.097
	F6 - Front leg - average force sagittal	0.026	[0.022 0.030]	12.402	<0.001
	F7 - Rear leg - push-off direction	0.010	[0.006 0.015]	4.982	<0.001
Adj. R² = 0.86					
response:	NAHBP				
predictor(s):	F1 - Rear leg - force sagittal	0.040	[0.036 0.045]	18.732	<0.001
	F3 - Front leg - push-off direction	0.032	[0.028 0.036]	14.854	<0.001
	F4 - Front leg - max force sagittal	0.030	[0.025 0.034]	13.789	<0.001
	F6 - Front leg - average force sagittal	0.026	[0.022 0.030]	11.990	<0.001
	F7 - Rear leg - push-off direction	0.010	[0.006 0.015]	4.717	<0.001
Adj. R² = 0.86						

ml: mediolateral; max: maximal

**Fig 3:**
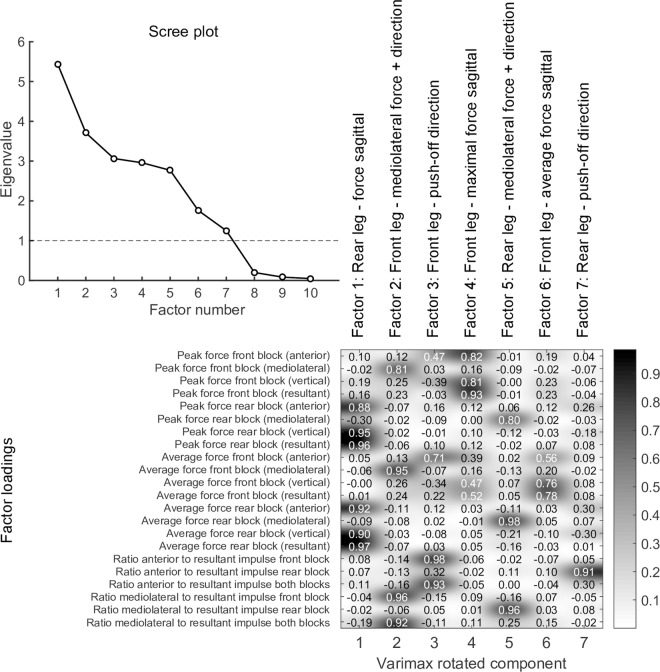
Factor analysis results.

**Fig 4:**
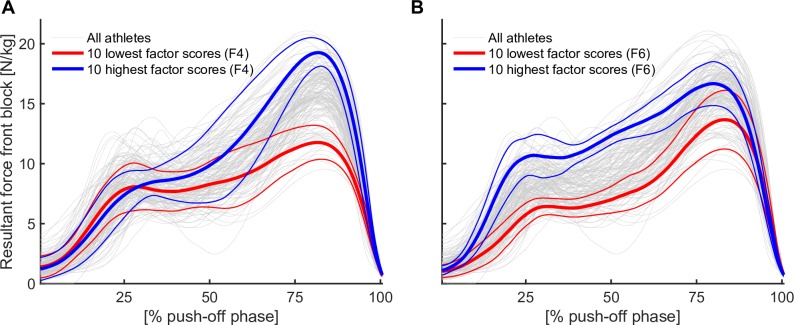
Comparison of the characteristics of factors 4 and 6. In A and B, the resultant force curves of the front block of athletes with the highest and lowest scores for factors 4 (A) and 6 (B) are visualized. Note the pronounced force peak at the end of the push-off phase for athletes scoring high on factor 4 (A), and the high force application in the early push-off phase for athletes scoring high on factor 6 (B).

The factor analysis model revealed that, based on both the Kaiser criterion (eigenvalue > 1) and by visual inspection of the scree plot (Fig 3), the first seven factors provide a sufficient representation of the force-application characteristics of NASs, across a wide range of overall sprint performance levels. After varimax rotation, the following interpretation was made based on analysis of the factor-loading structure (Fig 3): Variables associated with force application to the rear block and front block in the sagittal plane of motion were highly loaded on factors 1, 4 and 6 (eigenvalues after rotation, 5.4, 3.0 and 1.8), respectively (Fig 3), which were thus considered to represent underlying factors affecting the forces applied to propel the athlete forward out of the blocks. Parameters related to force application to the front block were highly loaded on factors 4 and 6, but in a different manner for each factor. High factor-4 scores were correlated with high peak force application that was concentrated at the end of the push-off phase after a moderate initial rise in force (Fig 4A). Parameters associated with high average force application, not necessarily with a high peak force but with a pronounced rise in force at the beginning of the push-off phase were more strongly loaded on factor 6 (Fig 4B). Fig 4 visualizes the differences between the two factors by showing the resultant front-block force-application waveforms of athletes with the ten highest and lowest scores for factors 4 and 6, respectively.

Mediolateral force application in the front and rear blocks and the corresponding mediolateral push-off directions loaded high on factors 2 and 5 (eigenvalues after rotation, 3.7 and 2.8), respectively (Fig 3). Parameters describing the direction of forces applied in the sagittal plane (RHRI) of the front and rear blocks loaded high on factors 3 and 7 (eigenvalues after rotation, 3.1 and 1.3), respectively (Fig 3). Therefore, we interpreted this factor as the ability to apply forces in the desired horizontal (running) direction in the sagittal plane.

When fitting linear regression models using the scores of the first seven varimax-rotated factors, an adjusted R² value of 0.86 was obtained (Table 2, middle). This model did not include interaction terms, but it explained only 1% less of the variance when compared to a model that included interaction terms. Furthermore, we found that a reduced model including only five factors as predictors achieved the same adjusted R^2^ values as the complete model including all seven factors (Table 2, bottom and middle). The highest coefficient was estimated for factor 1, which represents the amplitude of the forces applied to the rear block. Coefficients of factors 3, 4 and 6, which describe the force amplitude and direction were all similar (0.026–0.032), highlighting the similarity of their influence on overall SP. Interestingly, in the rear blocks, the coefficient for force amplitude (Factor 1) had a substantially higher coefficient estimate versus factor 7 (0.040 vs. 0.010), which describes push-off direction from the rear block. Therefore, it can be concluded that in the rear block, the strength of the push-off is more important than its direction.

**Fig 5:**
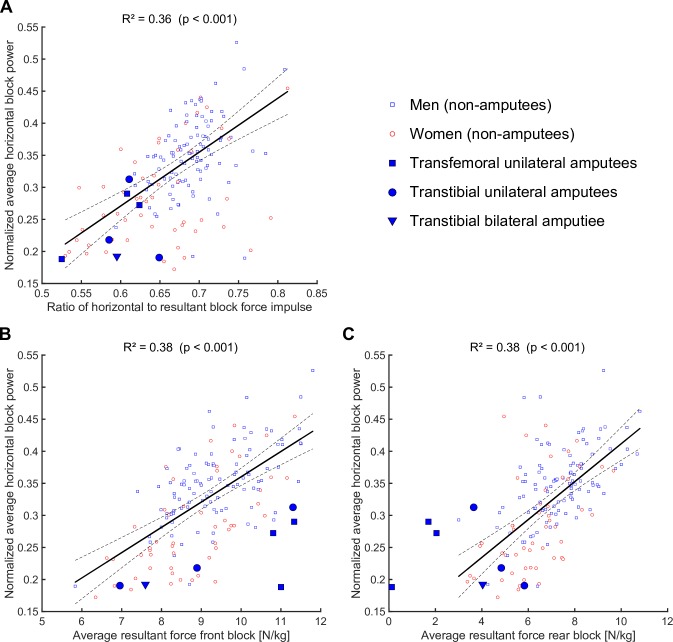
Scatter plots including fitted linear regression lines. Regression models were fitted using the non-amputee (NAS) data only. Regression lines are plotted along with broken lines that represent the 95% confidence interval.

Bivariate relationships between selected parameters and SP including the individual values of ASs are visualized in Fig 5.

Start performance was 33.8% lower (p<0.001) in ASs versus NASs matched with respect to relative 100 m PR (Table 3). When matched based on absolute 100 m PRs, a smaller and almost significant (*p* = 0.08) reduction of 17.7% was observed (Table 4). Force application to the rear block differed significantly between ASs and NASs in the sagittal plane (Tables 3 and 4), while effects were greater when subjects were matched with respect to their relative PRs. The direction of force application was more vertical for amputee athletes, particularly in the front block. Block times were significantly increased by 23.9% and 9.6% for ASs when compared to relative and absolute PR matched NASs, respectively (Tables 3 and 4).

**Table 3:** Comparison between non-amputee and amputee sprinters matched with respect to their relative 100 m personal records (PRs). In all unilateral amputees, the rear leg was the affected side. Values are presented in terms of the mean ± *SD*, 95% confidence interval of the difference between means, *p* values and effect sizes (Cohen’s *d*). Bold rows indicate a significant difference for this parameter (*p* < 0.05).

	Matching			All			95%					Effect size
	non-amputee			amputee			confidence					Cohen's
	athletes			athletes			interval				p-value	d
**Block time (s)**	**0.35**	**±**	**0.03**	**0.43**	**±**	**0.03**	**[**	**-0.11**	**-0.05**	**]**	**<0.001**	**3.03**
Hor. CoM velocity (m/s)	3.21	±	0.26	2.93	±	0.27	[	-0.01	0.56	]	0.056	1.04
**NAHBP**	**0.36**	**±**	**0.06**	**0.24**	**±**	**0.05**	**[**	**0.07**	**0.17**	**]**	**<0.001**	**2.11**
**Max. anterior force front (N/kg)**	**10.89**	**±**	**1.35**	**9.04**	**±**	**1.57**	**[**	**0.24**	**3.47**	**]**	**0.029**	**1.32**
Max. mediolateral force front (N/kg)	1.10	±	0.47	1.57	±	0.50	[	-0.99	0.05	]	0.071	0.99
Max. vertical force front (N/kg)	11.95	±	2.03	###	±	1.43	[	-1.80	1.43	]	0.806	0.10
Max. resultant force front (N/kg)	16.18	±	2.27	###	±	2.02	[	-1.16	3.15	]	0.332	0.45
**Max. anterior force rear(N/kg)**	**12.03**	**±**	**1.46**	**4.46**	**±**	**2.50**	**[**	**5.05**	**10.09**	**]**	**<0.001**	**4.26**
**Max. mediolateral force rear (N/kg)**	**-0.76**	**±**	**0.32**	**###**	**±**	**0.14**	**[**	**-0.77**	**-0.37**	**]**	**<0.001**	**1.98**
**Max. vertical force rear(N/kg)**	**9.62**	**±**	**1.61**	**4.22**	**±**	**2.67**	**[**	**2.71**	**8.10**	**]**	**0.002**	**2.80**
**Max. resultant force rear (N/kg)**	**15.41**	**±**	**2.04**	**6.15**	**±**	**3.59**	**[**	**5.64**	**12.87**	**]**	**<0.001**	**3.68**
Avg. anterior force front (N/kg)	6.41	±	0.91	5.71	±	0.97	[	-0.30	1.71	]	0.147	0.76
Avg. mediolateral force front (N/kg)	0.46	±	0.37	0.81	±	0.42	[	-0.78	0.09	]	0.105	0.91
Avg. vertical force front (N/kg)	6.73	±	1.10	7.69	±	1.48	[	-2.47	0.55	]	0.182	0.80
Avg. resultant force front (N/kg)	9.38	±	1.35	9.70	±	1.72	[	-2.08	1.44	]	0.688	0.22
**Avg. anterior force rear(N/kg)**	**5.70**	**±**	**0.94**	**2.14**	**±**	**1.22**	**[**	**2.32**	**4.81**	**]**	**<0.001**	**3.51**
Avg. mediolateral force rear (N/kg)	-0.11	±	0.28	0.01	±	0.11	[	-0.28	0.05	]	0.156	0.47
**Avg. vertical force rear(N/kg)**	**5.06**	**±**	**1.03**	**2.24**	**±**	**1.42**	**[**	**1.38**	**4.26**	**]**	**0.002**	**2.47**
**Avg. resultant force rear (N/kg)**	**7.81**	**±**	**1.29**	**3.18**	**±**	**1.83**	**[**	**2.77**	**6.49**	**]**	**<0.001**	**3.21**
**Ratio anterior / resultant Impulse front**	**0.69**	**±**	**0.04**	**0.59**	**±**	**0.04**	**[**	**0.05**	**0.13**	**]**	**<0.001**	**2.53**
Ratio anterior / resultant Impulse rear	0.73	±	0.03	0.62	±	0.21	[	-0.11	0.32	]	0.263	0.97
**Ratio anterior / resultant Impulse both**	**0.70**	**±**	**0.03**	**0.60**	**±**	**0.04**	**[**	**0.06**	**0.14**	**]**	**<0.001**	**3.27**
Ratio mediolateral / resultant Impulse front	0.05	±	0.04	0.08	±	0.03	[	-0.07	0.00	]	0.065	0.87
Ratio mediolateral / resultant Impulse rear	-0.01	±	0.04	###	±	0.21	[	-0.15	0.27	]	0.504	0.56
**Ratio mediolateral / resultant Impulse both**	**0.03**	**±**	**0.03**	**0.07**	**±**	**0.04**	**[**	**-0.08**	**-0.01**	**]**	**0.023**	**1.50**
Mass(kg)	76.09	±	7.66	###	±	6.90	[	-8.99	5.69	]	0.631	0.22
**Age (yrs)**	**21.47**	**±**	**4.08**	**###**	**±**	**3.07**	**[**	**####**	**-4.13**	**]**	**<0.001**	**1.95**
Height (m)	1.82	±	0.07	1.89	±	0.07	[	-0.15	0.00	]	0.054	1.07
**100 m PR (s)**	**10.54**	**±**	**0.54**	**###**	**±**	**0.31**	**[**	**-2.07**	**-1.32**	**]**	**<0.001**	**3.44**
Rel. 100 m PR (%)	110.0	±	5.66	###	±	5.92	[	-5.33	6.99	]	0.771	0.14

Matching non-amputee sample included 19 male athletes. Avg.: Average; Max.: Maximum; Small effect (d ≥ 0.2 and d < 0.5); Medium effect (d ≥ 0.5 and d < 0.8); Large effect (d ≥ 0.5)

**Table 4:** Comparison between non-amputee and amputee sprinters matched with respect to their absolute 100-m personal records (PRs). In all unilateral amputees, the rear leg was the affected side. Values are presented in terms of the mean ± *SD*, 95% confidence interval of the difference between means, *p* values and effect sizes (Cohen’s d). Bold printed rows indicate a significant difference for this parameter (*p* < 0.05).

	Matching			All			95%					Effect size
	non-amputee			amputee			confidence					Cohen's
	athletes			athletes			interval				p-value	d
**Block time (s)**	**0.40**	**±**	**0.03**	**0.43**	**±**	**0.03**	**[**	**-0.07**	**-0.01**	**]**	**0.025**	**1.15**
Hor. CoM velocity (m/s)	3.02	±	0.35	2.93	±	0.27	[	-0.22	0.40	]	0.548	0.27
NAHBP	0.29	±	0.07	0.24	±	0.05	[	-0.01	0.11	]	0.076	0.79
Max. anterior force front (N/kg)	10.18	±	1.59	9.04	±	1.57	[	-0.53	2.81	]	0.161	0.72
Max. mediolateral force front (N/kg)	1.18	±	0.44	1.57	±	0.50	[	-0.91	0.13	]	0.126	0.86
Max. vertical force front (N/kg)	11.76	±	2.50	12.14	±	1.43	[	-2.20	1.45	]	0.673	0.17
Max. resultant force front (N/kg)	15.66	±	2.52	15.19	±	2.02	[	-1.79	2.73	]	0.664	0.20
**Max. anterior force rear(N/kg)**	**8.21**	**±**	**1.66**	**4.46**	**±**	**2.50**	**[**	**1.21**	**6.29**	**]**	**0.009**	**1.93**
**Max. mediolateral force rear (N/kg)**	**-0.52**	**±**	**0.37**	**-0.19**	**±**	**0.14**	**[**	**-0.56**	**-0.10**	**]**	**0.008**	**1.03**
**Max. vertical force rear(N/kg)**	**7.69**	**±**	**1.51**	**4.22**	**±**	**2.67**	**[**	**0.77**	**6.17**	**]**	**0.018**	**1.81**
**Max. resultant force rear (N/kg)**	**11.24**	**±**	**2.16**	**6.15**	**±**	**3.59**	**[**	**1.45**	**8.72**	**]**	**0.012**	**1.92**
Avg. anterior force front (N/kg)	5.79	±	1.11	5.71	±	0.97	[	-0.97	1.14	]	0.862	0.08
Avg. mediolateral force front (N/kg)	0.48	±	0.36	0.81	±	0.42	[	-0.77	0.11	]	0.124	0.87
Avg. vertical force front (N/kg)	6.60	±	1.12	7.69	±	1.48	[	-2.62	0.43	]	0.138	0.89
Avg. resultant force front (N/kg)	8.92	±	1.26	9.70	±	1.72	[	-2.54	0.98	]	0.340	0.56
**Avg. anterior force rear(N/kg)**	**4.15**	**±**	**0.76**	**2.14**	**±**	**1.22**	**[**	**0.77**	**3.24**	**]**	**0.006**	**2.19**
Avg. mediolateral force rear (N/kg)	-0.07	±	0.28	0.01	±	0.11	[	-0.26	0.09	]	0.325	0.35
**Avg. vertical force rear(N/kg)**	**4.29**	**±**	**0.80**	**2.24**	**±**	**1.42**	**[**	**0.63**	**3.48**	**]**	**0.011**	**2.02**
**Avg. resultant force rear (N/kg)**	**6.12**	**±**	**1.02**	**3.18**	**±**	**1.83**	**[**	**1.09**	**4.79**	**]**	**0.007**	**2.25**
**Ratio anterior / resultant Impulse front**	**0.65**	**±**	**0.08**	**0.59**	**±**	**0.04**	**[**	**0.01**	**0.11**	**]**	**0.030**	**0.87**
Ratio anterior / resultant Impulse rear	0.68	±	0.04	0.62	±	0.21	[	-0.16	0.27	]	0.564	0.45
**Ratio anterior / resultant Impulse both**	**0.66**	**±**	**0.06**	**0.60**	**±**	**0.04**	**[**	**0.01**	**0.10**	**]**	**0.018**	**1.01**
Ratio mediolateral / resultant Impulse front	0.05	±	0.04	0.08	±	0.03	[	-0.07	0.01	]	0.104	0.80
Ratio mediolateral / resultant Impulse rear	-0.01	±	0.05	-0.07	±	0.21	[	-0.15	0.27	]	0.492	0.53
Ratio mediolateral / resultant Impulse both	0.04	±	0.03	0.07	±	0.04	[	-0.07	0.00	]	0.054	1.12
**Mass(kg)**	**63.28**	**±**	**7.54**	**77.74**	**±**	**6.90**	**[**	**####**	**-7.00**	**]**	**0.001**	**1.96**
**Age (yrs)**	**19.00**	**±**	**3.69**	**29.00**	**±**	**3.07**	**[**	**####**	**-6.60**	**]**	**<0.001**	**2.84**
**Height (m)**	**1.74**	**±**	**0.08**	**1.89**	**±**	**0.07**	**[**	**-0.23**	**-0.07**	**]**	**0.002**	**1.82**
100 m PR (s)	12.21	±	0.36	12.24	±	0.31	[	-0.36	0.31	]	0.865	0.08
**Rel. 100 m PR (%)**	**119.1**	**±**	**3.57**	**109.2**	**±**	**5.92**	**[**	**3.91**	**15.90**	**]**	**0.005**	**2.26**

Matching non-amputee sample included 16 athletes (4 males, 12 females). Avg.: Average; Max.: Maximum; Small effect (d ≥ 0.2 and d < 0.5); Medium effect (d ≥ 0.5 and d < 0.8); Large effect (d ≥ 0.5)

**Fig 6:**
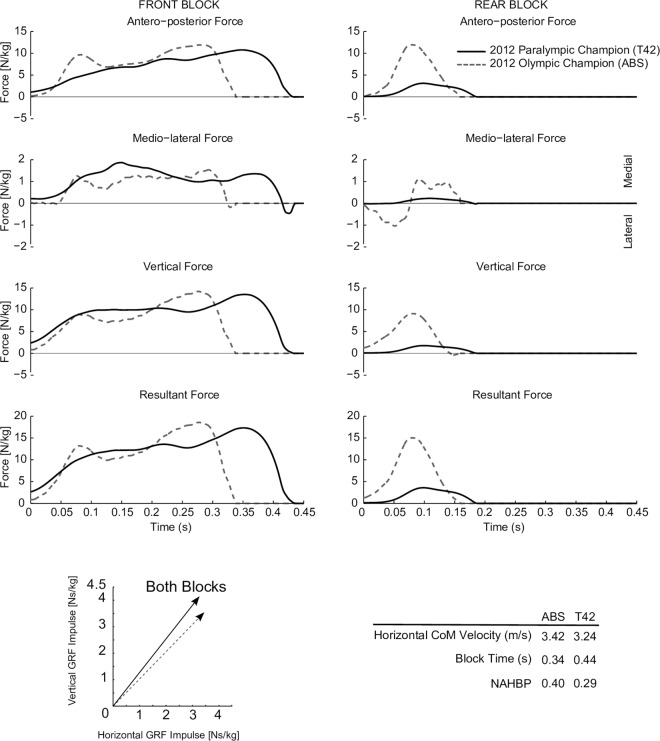
Representative waveform data from the best non-amputee and the best transfemoral unilateral amputee in the study. Block times are clearly elongated in the front leg, while force application is substantially reduced for the rear (affected) leg of the transfemoral amputee athlete. Push-off angle is more vertically oriented in the amputee athlete.

Differences in force-application patterns were clearly seen when the force waveforms of the best (with respect to 100 m PR) NAS and the best unilateral transfemoral amputee were compared (Fig 6).

**Fig 7:**
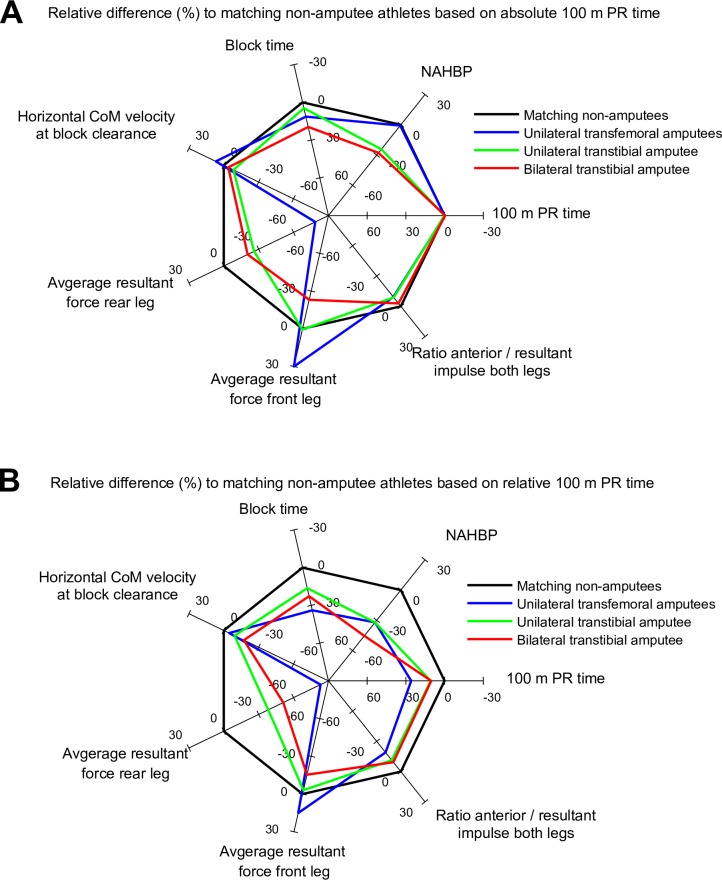
Radar chart representation of the relative difference between amputee athletes and matching controls. Here, controls were matched with respect to the individual’s amputation level. For each amputee athlete, three non-amputee athletes were matched with respect to (A) their absolute 100 m personal record (PR) or (B) their 100 m PR relative to the respective 100 m world record (for the corresponding amputation level). The results are displayed as relative differences (%) with respect to the matching controls. Positive axis directions were defined such that better performances in a certain parameter are outside of the zero difference (non-amputee) line and worse performances are inside that line.

When comparing athletes with different amputation levels, it was seen that athletes with more proximally (higher) located amputations exerted less force with their affected limb than athletes with a more distal (lower) amputation or a bilateral amputation (Fig 7). Nonetheless, the unilateral transfemoral (proximal) amputees analyzed in the present study compensated for this deficiency by applying a proportionally greater force with the (non-affected) front leg, thereby achieving a better overall SP than transtibial (distal) amputees.

## Discussion

The first task of the present study was to investigate the presence of a potentially underlying factor structure for ground-force application during the sprint start. The present NAS dataset was very well suited for such an analysis as it was sufficiently large and represented 100 m sprinters from all relevant performance categories, including even the highest level of performance. Although seven factors seem to be a good choice for proper representation of the variability contained in the ground-force application data set based on the Kaiser and elbow criteria, the results of the present study indicate that only five factors are needed to explain 86% of the variance observed in SP. The remaining two factors, which are related to force application in the mediolateral direction, did not significantly improve the predictive power of any of our multiple linear regression models. This suggests that no performance benefit would accrue from modifying a non-amputee’s starting-technique to minimize mediolateral-force application and achieve a straighter push-off in the forward direction.

Using the scores from the above explorative factor analysis as predictors in a multiple regression analysis offers two main advantages over the more common practice of implementing all directly measured parameters in the multiple regression analysis: On the one hand, when estimating the coefficients of the regression model, it avoids potential problems that can result from multicollinearity among the predictor variables [23]. In the present study, multicollinearity was clearly evident among the force-application parameters. This outcome is understandable since many of these parameters (e.g. for different force components) cannot be considered independent of each other, as they are the product of the same biomechanical action. On the other hand, absolute values of the estimated coefficients in the regression model can be directly compared to evaluate their importance. This is because the original parameters were standardized to have a mean of 0 and a standard deviation of 1 before being used in the factor-analysis calculations. As a result, the importance of each latent factor for the prediction of start performance can be directly derived from the coefficients of the regression model.

In the present study, the factor representing force application to the rear block showed the highest estimated coefficient (0.040; factor 1), followed by those representing push-off direction in the sagittal plane (0.032; factor 3) and force application to the front block (0.030 and 0.026; factors 4 and 6). The 95% confidence intervals of factor 1 overlap minimally with those of the other factors, indicating that it is potentially more important for SP than the other factors. This highlights the importance of high force application to the blocks in the sprint start. High coefficient estimates were also found for factors representing the amplitude and direction of force application to the front block. With respect to starting technique, this indicates that a high average force needs to be applied to both blocks in the horizontal direction. It is interesting to note that factors relating to force-application amplitude and direction were of similar magnitude in the front block, whereas in the rear block, the coefficient estimate for the direction factor (0.010; factor 7) was 4-fold lower than the corresponding amplitude coefficient (0.040; factor 1). This result indicates that forces at the rear block need to be maximised, but ensuring that they are well aligned to the running direction is less important. In contrast, at the front block, both force amplitude and direction were of similar importance.

Another interesting result of the present study is the fact that two factors (4 and 6) were related to the force amplitude at the front block. As these factors are independent of each other, they may represent two different targets for improving SP. Looking at Fig 4, we can see that athletes scoring differently on factors 4 and 6 used different strategies for resultant-force application. Those with high factor-4 scores exerted substantially more force (about double) towards the end of the push-off versus the onset. In contrast, although athletes with high factor-6 scores also peaked towards the end, relative to high factor-4 athletes they pushed harder early on and less at the end, thereby producing a more even distribution of force over the duration of the push-off. The latter strategy for maximising the resultant impulse appears to focus less on achieving a high peak force and more on attaining high amplitudes in the first half of the push-off. Future studies should investigate these strategies in greater depth, in order to establish whether they are the result of different technical models of athletes and how they are related to the block distance, distance to the starting line and other parameters related to starting technique.

If instrumented starting blocks are available for performance diagnostics or for biofeedback training, we recommend use of at least the average resultant forces in the front and the rear blocks, as well as the ratio of the anterior and resultant impulses as criterion parameters, since they were highly loaded on most of the factors important for prediction of SP. As mediolateral-force application parameters did not improve the prediction of starting performance, they appear to be of less importance for these tasks. The design of instrumented starting blocks for performance diagnostics or biofeedback might therefore be simplified by using only 2D force sensors to measure forces in the running and vertical directions.

Once our exploratory analysis of the NAS data was complete, we compared the start performance of ASs and NASs. All unilateral ASs preferred to place their affected leg on the rear block, which is consistent with observations from video recordings summarized in Taboga et al. [7]; 86% of unilateral ASs utilized this pattern of leg placement in the 2012 Paralympic Games. The results of the present study show that force application to the blocks is clearly impaired in ASs using sprint-specific prostheses, which is in line with the data of Taboga et al. [7]. This impairment was higher for athletes with above-knee amputations (versus below-knee amputees), but the difference was not statistically assessed owing to the low sample sizes. When considering the fact that force application to rear block (factor 1) is more important than for the front block, it is interesting to note that most ASs prefer putting their affected legs in the rear block. Still, the factor analysis revealing the importance of rear block force application was performed within the NAS dataset only. Therefore, any inference from these results to a NAS population might be invalid. Other factors, like dynamic stability or the performance in subsequent steps might be more important in ASs and might therefore have a stronger influence on their foot placement strategy. Future research on a bigger ASs sample needs to identify the underlying mechanisms responsible for start performance in a similar way as it has been achieved for NASs in the present study.

Comparing ASs and NASs that were matched with respect to their relative (to corresponding world record) 100 m PRs, SP was reduced by 33.8% for the amputee athletes, and this was associated with an average increase in block time of 0.08 s. Interestingly, average resultant forces applied to the front blocks were not lower in ASs, but they were applied in a more vertical direction, which has been shown to be detrimental for acceleration performance [8, 9]. Furthermore, forces in the front block were applied in a more laterally oriented fashion by the ASs, which might be a consequence of the specific requirements put upon transfemoral amputees when swinging the rear leg forwards after leaving the blocks. Because they are not capable of actively achieving and maintaining a flexed knee angle by means of hamstring and/or gastrocnemius force generation, they are required to rotate their affected leg laterally in order to avoid contacting the ground with the prosthesis.

When the two groups were matched with respect to their absolute 100 m PRs, start performance was again reduced, this time by 17.7%—however, with a *p* value of 0.08, this reduction was not sufficient to be considered statistically significant. Both groups of athletes were similar with respect to their overall 100 m race performance (100 m PR). From these results, it can be concluded that during the race phases where speed was constant, ASs must have performed better compared to their non-amputee counterparts. We suggest two possible explanations for this. Firstly, the level of professionalism may be significantly higher for the particular ASs in this study. Some of these athletes compete at the very highest level of their sport, in the 100 m, 200 m and long jump, and this is reflected in the significant difference for relative 100 m PRs (109% vs. 119%, respectively). These amputee athletes may simply spend, on average, more time and effort on training and active recovery than the matching NASs. The second possible explanation is that sprint-specific prostheses, though inferior during the start phase, performed better in replacing the functionality of biological limbs during the maximum-constant-speed phase of the race by enabling the spring-like energy exchange in the lower limbs during ground contact [6]. Additionally, they might be allowing for a more rapid limb-swing motion owing to their low mass and moment of inertia [24]. During the start and early acceleration phases of a 100 m race, the majority of the mechanical work is performed by the contractile components of the muscle–tendon-units, but as the race goes on, the contribution of passive elastic structures, like tendons and ligaments, becomes dominant [25]. In amputee athletes, the ratio of passive elastic structures to active contractile muscle mass is higher than in non-amputee athletes, which makes their legs better suited to constant speed running than to accelerating.

Nevertheless, the interaction between passive prosthetics and the remaining limb anatomy is challenging from a coordinative perspective, not least on account of the missing sensory input from muscle spindles, Golgi tendon organs and other biological sensors at distal locations on the leg. Furthermore, it has been argued that maximum sprint velocity can be impaired by limitations that RSPs impose on ground-force application and leg stiffness [26, 27].

In summary, the results of the present study emphasize the importance of high average force application to both rear and front blocks. In addition, the forces should be applied as horizontally as possible, in the direction of forward motion. The avoidance of high mediolateral forces had no significant effect on start performance in non-amputee sprinters. These features of successful push-off from the starting blocks are consistent with recently published studies of world-class athletes [28]. Force application to the starting blocks was clearly impaired in amputees using RSPs (versus non-amputees), with greater impairment occurring in athletes with more proximal amputations (higher up leg). This impairment led to significantly reduced start performance in the amputee sprinters. On the other hand, their RSPs appear to better replicate the functionality of biological limbs during the constant-speed phases of the 100-m race.
